# Fluorophore Absorption Size Exclusion Chromatography (FA-SEC): An Alternative Method for High-Throughput Detergent Screening of Membrane Proteins

**DOI:** 10.1371/journal.pone.0157923

**Published:** 2016-06-22

**Authors:** Sung-Yao Lin, Xing-Han Sun, Yu-Hsuan Hsiao, Shao-En Chang, Guan-Syun Li, Nien-Jen Hu

**Affiliations:** Institute of Biochemistry, National Chung Hsing University, Taichung, 40227, Taiwan, ROC; Griffith University, AUSTRALIA

## Abstract

Membrane proteins play key roles in many fundamental functions in cells including ATP synthesis, ion and molecule transporter, cell signalling and enzymatic reactions, accounting for ~30% genes of whole genomes. However, the hydrophobic nature of membrane proteins frequently hampers the progress of structure determination. Detergent screening is the critical step in obtaining stable detergent-solubilized membrane proteins and well-diffracting protein crystals. Fluorescence Detection Size Exclusion Chromatography (FSEC) has been developed to monitor the extraction efficiency and monodispersity of membrane proteins in detergent micelles. By tracing the FSEC profiles of GFP-fused membrane proteins, this method significantly enhances the throughput of detergent screening. However, current methods to acquire FSEC profiles require either an in-line fluorescence detector with the SEC equipment or an off-line spectrofluorometer microplate reader. Here, we introduce an alternative method detecting the absorption of GFP (FA-SEC) at 485 nm, thus making this methodology possible on conventional SEC equipment through the in-line absorbance spectrometer. The results demonstrate that absorption is in great correlation with fluorescence of GFP. The comparably weaker absorption signal can be improved by using a longer path-length flow cell. The FA-SEC profiles were congruent with the ones plotted by FSEC, suggesting FA-SEC could be a comparable and economical setup for detergent screening of membrane proteins.

## Introduction

Membrane proteins are abundant in cells and play pivotal roles in solute homeostasis, signal transduction and energy production. It is estimated that ~30% of the genome encodes integral membrane proteins. However, despite the first crystal structure of a membrane protein was solved in 1985 [1], to date, there are only ~600 crystal structures of unique membrane proteins, accounting for only 1.5% of total deposits in Protein Data Bank (refer to Stephen White’s database http://blanco.biomol.uci.edu/mpstruc/). Although protein samples are no longer purified from natural sources but over-expressed as recombinant proteins in bacterial, yeast, insect and mammalian cells, the number of determined membrane protein structures still lags behind soluble proteins. The major obstacles of obtaining membrane protein structures at atomic level are their low heterologous expression level, poor protein stability in detergent micelles and difficulty in crystallization. As a result, it requires a time-consuming and laborious process to perform a series of empirical screenings, such as expression conditions, homolog proteins, detergents, additives and conformationally sensitive antibodies, to eventually obtain crystals of membrane proteins with reasonable diffraction qualities.

In early 2000, Drew and colleagues developed the GFP-based fusion technology to assist in the survey stages of membrane protein preparation. The C-terminally fused GFP serves as a folding indicator since it can fold properly only if the upstream membrane protein inserts into the membrane [2, 3]. By these means, one can not only correlate the whole-cell fluorescence count with the expression of correctly integrated membrane proteins, but also examine the size of overexpressed membrane proteins using in-gel fluorescence instead of Western blot. Using the GFP-based technology as a starting point, fluorescence-detection size-exclusion chromatography (FSEC) [4] was developed by coupling an in-line fluorescence detector to an HPLC system. This methodology allowed the evaluation of monodispersity of membrane protein samples in detergent micelles using solubilized material from whole cell lysates or crude membrane. Similar to size-exclusion chromatography (SEC), in which folded and stable protein samples exist as monodisperse species in solution and normally exhibit a symmetrical Gaussian curve, FSEC monitors the molecular distribution of GFP-fused membrane proteins in a protein-detergent complex (PDC) without the need for prior purification. Furthermore, only nanogram quantities of non-purified protein sample are required for the assay. The technology significantly improves the throughput of “pre-crystallization screening” in the process of membrane protein structure determination.

GFP-based expression screening and FSEC have been widely applied in prokaryotic and eukaryotic expression systems for heterologous membrane protein production, including *E*. *coli* [4], *Saccharomyces cerevisiae* [5, 6], *Pichia pastoris* [7, 8], insect cells [9, 10] and human cells [4]. In recent years, modified strategies have been developed on the basis of FSEC for various specific experimental conditions. For example, Hu et al. [10] published a high-throughput screening method describing the protocol of expression and stability screening for eukaryotic membrane proteins using a pTriEx-based vector containing promoter components for *E*. *coli*, insect cells and mammalian cells allowing multi-host screen. Gouaux and coworkers [11] developed an FSEC-based thermostability assay (FSEC-TS) analyzing the FSEC profiles of heated protein samples to screen the thermostability of eukaryotic membrane proteins in different ligands, ions and lipid derivatives. Backmark et al. [12] synthesized a fluorescent NTA probe that binds the polyhistidine tags of membrane proteins, by which one can conduct FSEC without GFP fusion because GFP may cause complication in folding and functionality of target proteins. Parcej et al. [13] developed multicolor (MC)-FSEC by constructing two plasmids carrying mVenus and mCeruelan respectively for co-expression. This method enables identification of correct assembly as well as stoichiometry of hetero-oligomeric membrane protein complexes.

Conventionally, FSEC is performed on an HPLC system directly coupled to a fluorescence detector [4]. In some laboratories, the protein samples are solubilized in different detergents and loaded via an autosampler into a UHPLC system, with FSEC data collection over night [10]. Alternatively, FSEC profiles can also be traced by plotting the fluorescence intensities in each SEC-fractionized wells using a 96-well microplate spectrofluorometer against the fraction numbers [5, 6]. The former option significantly enhances the throughput of screening process but requires an in-line fluorimeter connected to the HPLC system. However, high costs and instrument compatibility are major concerns. The latter option is labor-intensive because the 96-well plates containing the chromatography fractions need to be delivered to a microplate reader, thus making this method incompatible with the HPLC autosampler. Additionally, the smoothness of FSEC profile using the latter option is poorer than the former setup.

The EGFP encoded by pWaldo-GFPe has peak excitation and emission wavelengths at 485 and 512 nm, respectively [3, 14]. In order to allow GFP to emit fluorescence, light must be absorbed by EGFP in order for its transition to the excited state. We thus undertook to investigate the suitability of the absorption rather than the emission spectrum for SEC profile acquisition. Here, we present the Fluorophore Absorption SEC (FA-SEC) profiles of two integral membrane proteins: ASBT_NM_, a bacterial homolog of human Apical Sodium-dependent Bile acid Transporter from *Neisseria menigitidis* [15], and HiTehA, a bacterial homolog of plant SLAC1 anion channel from *Haemophilus influenza* [16]. We monitored the characteristic absorption of EGFP at 485 nm and plotted the FA-SEC profiles. The results revealed a linear correlation of absorption and fluorescence intensities for purified recombinant EGFP and detergent-solubilized membranes of EGFP-fused membrane proteins. We also demonstrated that the FA-SEC profiles are comparable with the FSEC profiles. This modified method provides an alternative approach to monitor the monodispersity and stability of EGFP-fused membrane proteins using an HPLC system equipped with a multiple wavelength absorption detector, which is more commonly found in research laboratories than in-line fluorescence spectrometers.

## Materials and Methods

### Plasmid Construction

For the production of recombinant EGFP, we constructed the pEGFP-His6 plasmid. The DNA fragment of EGFP was amplified by PCR with primers containing the *Nde*I and *Xho*I restriction sites using pWaldo-GFPe as the template, and subcloned into pET21a vector (Novagen). For the FA-SEC measurements, pASBT_NM_-EGFP-His8 and pHiTehA-EGFP-His8 were constructed individually using the vector pWaldo-GFPe [3, 14]. For the FA-SEC background profiles, the DNA fragments encoding EGFP was deleted from pASBT_NM_-EGFP-His8 and pHiTehA-EGFP-His8 using NEBuilder HiFi DNA Assembly Kit (New England BioLabs), producing pASBT_NM_-His8 and pHiTehA-His8, respectively.

### Purification of Recombinant EGFP-His6

pEGFP-His6 was transformed into *E*. *coli* BL21(DE3) and induced with 0.4 mM IPTG for over-expression. The cell pellet was resuspended in lysis buffer (1× PBS, protease inhibitor cocktail) followed by cell lysis using a cell disruptor (Constant System). The soluble part was fractionated by ultracentrifugation at 150,000 g for 10 min. The supernatant was subject to immobilised metal ion affinity chromatography (IMAC) with a Ni-NTA resin pre-equilibrated in the buffer containing 1xPBS and 20 mM imidazole. EGFP-His6 was eluted using buffer containing 1xPBS and 250 mM imidazole. The concentration of purified EGFP-His6 was determined by BCA assay (Bio-RAD). The fluorescence count was measured using a microplate spectrofluorometer (Tecan) (λ_ex_ = 485nm, λ_em_ = 512 nm).

### Preparation of Solubilized Crude Membranes

Expression of ASBT_NM_-EGFP-His8 and HiTehA-EGFP-His8 was performed as reported previously [15, 17]. Briefly, the target genes encoding ASBT_NM_ and HiTehA were cloned into the EGFP-His8 fusion vector pWaldo-GFPe individually [3] and over-expressed in *E*. *coli* C43(DE3) by adding 0.4 mM IPTG when OD(600 nm) reached 0.4. The temperature was lowered to 25°C after induction and the incubation continued overnight. Cell pellets were resuspended in lysis buffer and lysed using a cell disruptor (Constant System). After removing the unbroken cell debris at low speed (6,000 g, 10 min), the membrane fractions were isolated using ultracentrifugation (150,000 g for 45 min). For FA-SEC experiments, the isolated membranes were resuspended in 1× PBS buffer and the total protein concentration was adjusted to 8 mg ml^-1^ as measured using BCA assay.

### Purification of ASBT_NM_-EGFP-His8

ASBT_NM_-EGFP-His8 was purified as detailed previously [15]. Briefly, to solubilize ASBT_NM_-EGFP-His8, 40 ml of crude membranes (total protein concentration = 15 mg ml^-1^) were added to 180 ml of solubilization buffer containing 1xPBS, 100 mM NaCl, 10 mM imidazole, 10% glycerol and 1% DDM with gentle agitation for 1h at 4°C. The mixture was subject to ultracentrifugation (150,000 g for 1h) to remove non-solubilized material. ASBT_NM_-EGFP-His8 fusion protein was purified using Ni-NTA resin pre-equilibrated using IMAC buffer containing 1xPBS, 100 mM NaCl, 10 mM imidazole, 10% glycerol and 0.03% DDM. After thorough wash of 20 column-volume of IMAC buffer containing 30 mM imidazole, ASBT_NM_-EGFP-His8 was eluted using IMAC buffer containing 250 mM imidazole. For tagless ASBT_NM_, the C-terminal EGFP-His8 was cleaved using TEV protease and removed by reverse IMAC using His-TRAP hp column (GE) pre-equlibrated in the buffer containing 20 mM Tris (pH 7.5), 150 mM NaCl, 20 mM imidazole and 0.03% DDM [3].

### Fluorescence and Absorption Measurement of Purified ASBT_NM_-EGFP-His8

The correlation curve for absorption and fluorescence was measured using purified ASBT_NM_-EGFP-His8 at concentration of 0.1 mg ml^-1^. 1 ml of original and 2-fold serial-diluted fusion protein samples were subject to measurement of absorption at 485 nm using a UV spectrophotometer (GeneQuant). 100 μl of each serial-diluted samples were then transferred to a 96-well plate for fluorescence measurements (λ_ex_ = 485nm, λ_em_ = 512 nm) using a microplate spectrofluorometer (Tecan). Data were acquired in triplicate and statistically analyzed for mean and standard variation.

### FA-SEC Profiles Using Absorption Detector

540 μl of ASBT_NM_-EGFP-His8 or HiTehA-EGFP-His8 crude membranes prepared as mentioned above (total protein concentration = 8 mg ml^-1^) were mixed with 60 μl of detergent stock followed by gentle agitation for 1 hr at 4°C. The non-solubilized material was separated by ultracentrifugation at 150,000 g for 45 min. FA-SEC was performed by injecting 500 μl supernatant of the centrifugation step into a pre-packed Superose 6 10/300 GL column (GE) pre-equilibrated in SEC buffer (20 mM Tris [pH 7.5], 150 mM NaCl and 0.03% DDM), at flow rate of 0.5 ml min^-1^. The FA-SEC profile was recorded using the in-line UV-900 multiple-wavelength monitor (GE) by monitoring absorbance at 485 nm. Flow-cells of 2 and 10 mm path-lengths were both used to compare the absorption intensity. In this study, FA-SEC profiles were acquired using 10 mm flow-cells if no particular notice.

### FSEC Profiles Using Microplate Spectrofluorometer

The fractions (200 μl each) of the FA-SEC experiment were collected on a 96-well black-bottom microplate (Greiner). The fluorescence count of each sample in the microplate was then measured row-by-row (λ_ex_ = 485nm, λ_em_ = 512 nm) using a microplate spectrofluorometer (Tecan). The fluorescence counts were plotted against retention volume (available through conversion of the fraction number).

## Results

### The UV Absorption of ASBT_NM_-EGFP-His8 Is Linearly Correlated to Its Fluorescence Emission

**Fig 1:**
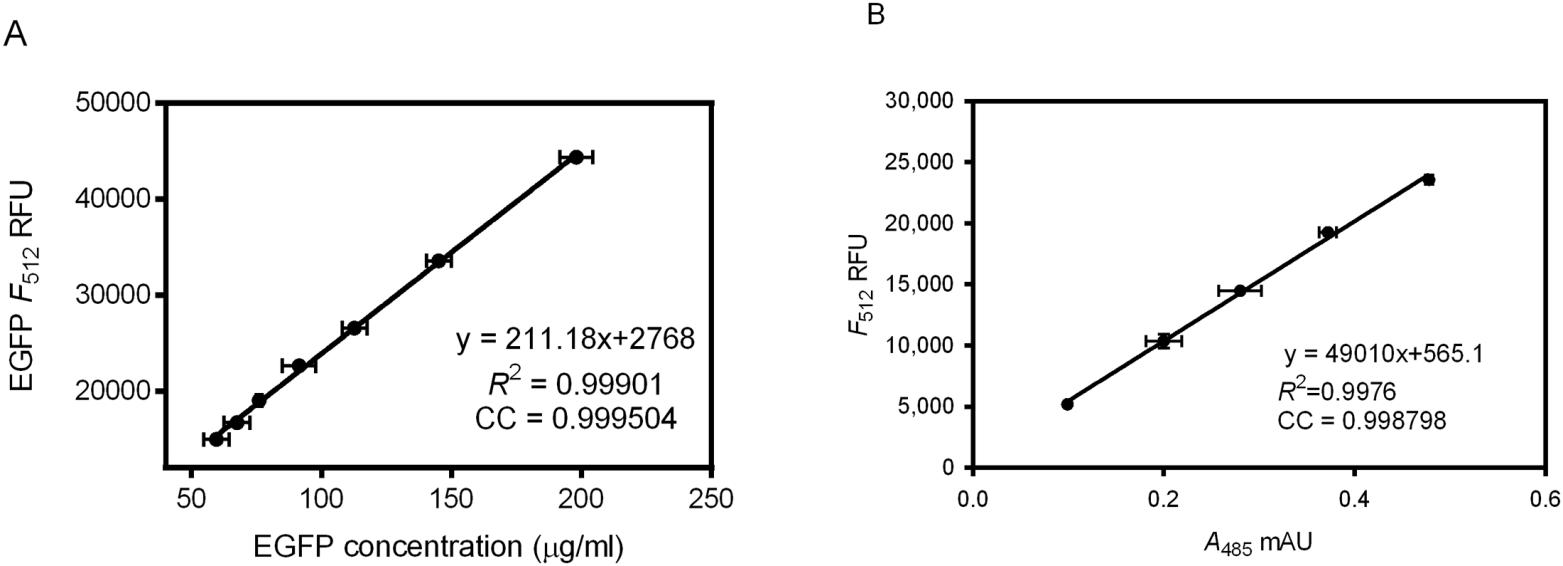
The correlation between the fluorescence and absorption of purified ASBT_NM_-EGFP-His8 fusion protein. (A) A standard curve of EGFP-Hi6 fluorescence emission (λ_ex_ = 485nm, λ_em_ = 512 nm) against EGFP-His6 concentration (mg/ml) determined by BCA assay. (B) A plot of purified ASBT_NM_-EGFP-His8 fluorescence emission (λ_ex_ = 485 nm, λ_em_ = 512 nm) against absorption at 485 nm. Each data point represents 0.1 mg ml^-1^ and 2-fold serial diluted ASBT_NM_-EGFP-His8 (from high to low concentrated). Vertical and horizontal error bars represent the standard error of the mean (n = 3) from three independent measurements.

The peak excitation and emission wavelengths for EGFP have been characterized according to a thorough study of fluorescent proteins [18]. For an optimum signal-to-noise ratio, excitation at 485 nm and emission at 512 nm were chosen due to lower background noise [5]. We first constructed a linear standard curve that defined the correlation of protein concentration and fluorescence emission of purified EGFP-His6 (Fig 1A). The result is in great agreement with previously published conversion factor [6]. This in-house standard enables quantification of protein concentration using fluorescence emission intensity.

For a given fluorophore, the number of absorbed photons is proportional to the number of emitted photons. We intended to validate the intensity correlation between absorption and emission of the EGFP fused to membrane proteins. In the experiment, the absorption of purified ASBT_NM_-EGFP-His8 was acquired using a spectrophotomer, while the fluorescence was acquired using a microplate spectrofluorometer (see Materials and Methods). The measurements using purified ASBT_NM_-EGFP-Hi8 fusion protein indicate a near-perfect positive correlation (c.c. = 0.99879) and excellent data fitting statistics (*R*^2^ = 0.9976) (Fig 1B). The correlation demonstrates that FA-SEC traces monitored at 485 nm delivers comparable results to the fluorescence profiles measured in traditional FSEC.

### The Sensitivity of FA-SEC Can Be Improved Using Longer Path-Length Flow Cell

**Fig 2:**
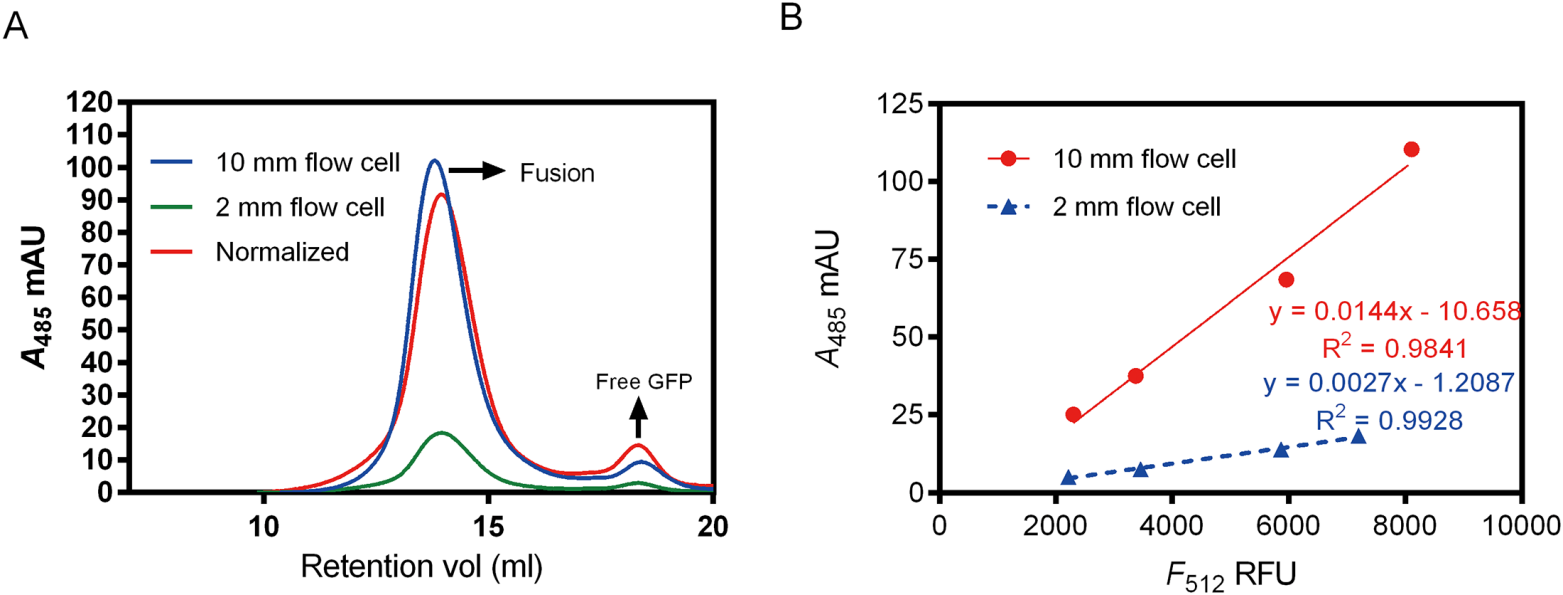
Signal enhancement of EGFP absorption using 10 mm path-length flow cell. (A) FA-SEC profiles of detergent solubilized membranes containing ~160 μg ASBT_NM_-EGFP-His8 recorded by 2 (green trace) and 10 mm (blue trace) path-length flow cells. The normalized profile (red trace) is plotted using the A485 in FA-SEC profile of 2 mm flow cell multiplied by 5. (B) Correlations of peak absorption (*A*_485_) and fluorescence (*F*_512_) acquired from the FA-SEC and FSEC profiles of different serial diluted ASBT_NM_-EGFP-His8 DDM-solubilized membranes (approximately containing 160, 80, 40, and 20 μg of ASBT_NM_-EGFP-His8 from high to low concentrated samples) injected in Seuperose 6 column, as exampled in Fig 3. The intensities of absorption maxima collected by 10 and 2 mm path-length flow cells are plotted as red and blue lines, respectively. Each data point represents different serial diluted detergent-solubilized crude membranes.

As standard flow cell path-lengths for major HPLC manufacturers are normally less than 5 mm, we intended to improve the signal-to-noise ratio by using longer path-length flow cell. We recorded the FA-SEC profiles of detergent solubilized membranes containing approximately 160 μg ASBT_NM_-EGFP-His8 using the in-line UV detector set at 485 nm. The absorption intensities measured at path-length of 2 and 10 mm respectively were then compared. The absorption profile measured by the 10 mm flow cell showed nearly identical retention volume (~14 ml) compared to the one measured by the 2 mm flow cell. However, the absorption peak height was roughly 5-fold increased (Fig 2A, blue and green traces). Normalizing the FA-SEC profile acquired from the 2 mm flow cell by multiplication factor of 5 indicates that the normalized profile is comparable to the profile measured by 10 mm flow cell, although the normalized profile shows lower intensity at the fusion peak but higher intensity at the free GFP peak (Fig 2A, red trace). It is also noticeable that the 2-fold serial diluted samples containing approximately 80, 40 and 20 μg of ASBT_NM_-EGFP-His8 (S1 Fig), still reveal a significant and readily observable absorption peak (A_485_ ~ 24 mAU) even in the most diluted sample (20 μg). However, signals for either sample were essentially undetectable when using a 2 mm flow cell, indicating that the significant improvement of the absorbance signal with the long path-length (10 mm) flow cell. Furthermore, the results obtained with the 10 mm cell were in good agreement with the normalized profiles (S1 Fig). An absorption-emission correlation analysis further demonstrates that the slope of fitted linear regression lines for the 10 mm flow cell (red line in Fig 2B, y = 0.0144x) is five-fold higher compared to the 2 mm one (blue line in Fig 2B, y = 0.0027x), as expected.

### Background A485 Signal Detected from the EGFP-Deleted Membrane Proteins

While developing the method, we examined whether intrinsic chromophores, such as hemes, may interfere the absorption at 485 nm. We first tested the DDM-solubilized membranes of IPTG induced *E*. *coli* C43(DE3) transformed with empty pET28a(+) vector, and a broad FA-SEC peak profile with poor symmetry and monodispersity was observed (S2 Fig). Surprisingly, injecting detergent-solubilized membranes containing EGFP-deleted ASBT_NM_-His8 or HiTehA-His8 revealed FA-SEC peaks with moderate monodispersity (S2 Fig). In comparison with ASBT_NM_-EGFP-His8 and HiTehA-EGFP-His8, the FA-SEC peaks of EGFP-deleted ASBT_NM_-His8 and HiTehA-His8 show great correspondence with the EGFP-containing counterparts in terms of retention volume, except the OG-solubilized membrane of ASBT_NM_ (S3 Fig). We also plotted the FA-SEC profile of purified ASBT_NM_, of which the EGFP and His-tag were cleaved by TEV protease and the background peak was almost invisible (S2 Fig), implicating the chromophores in the ASBT_NM_-His8 or HiTehA-His8 membranes are associated with the overexpressed membrane proteins via the C-terminal His-tag. It is thus speculated that the background A485 signal of empty pET28a(+) was probably contributed by the broad spectrum of heme-binding proteins, such as cytochromes, harbored abundantly in the native *E*. *coli* membranes [19]. Overexpression of heterogeneous membrane proteins may disrupt the synthesis of native membrane proteins and the nonspecific background absorption is thus suppressed. The A485 peaks observed in the ASBT_NM_-His8 or HiTehA-His8 FA-SEC profiles are presumably originate from the free heme molecules associated with the overexpressed proteins via heme iron coordinated to the octa-histidine tag [20, 21].

### FA-SEC Profiles Are Similar to FSEC Profiles

**Fig 3:**
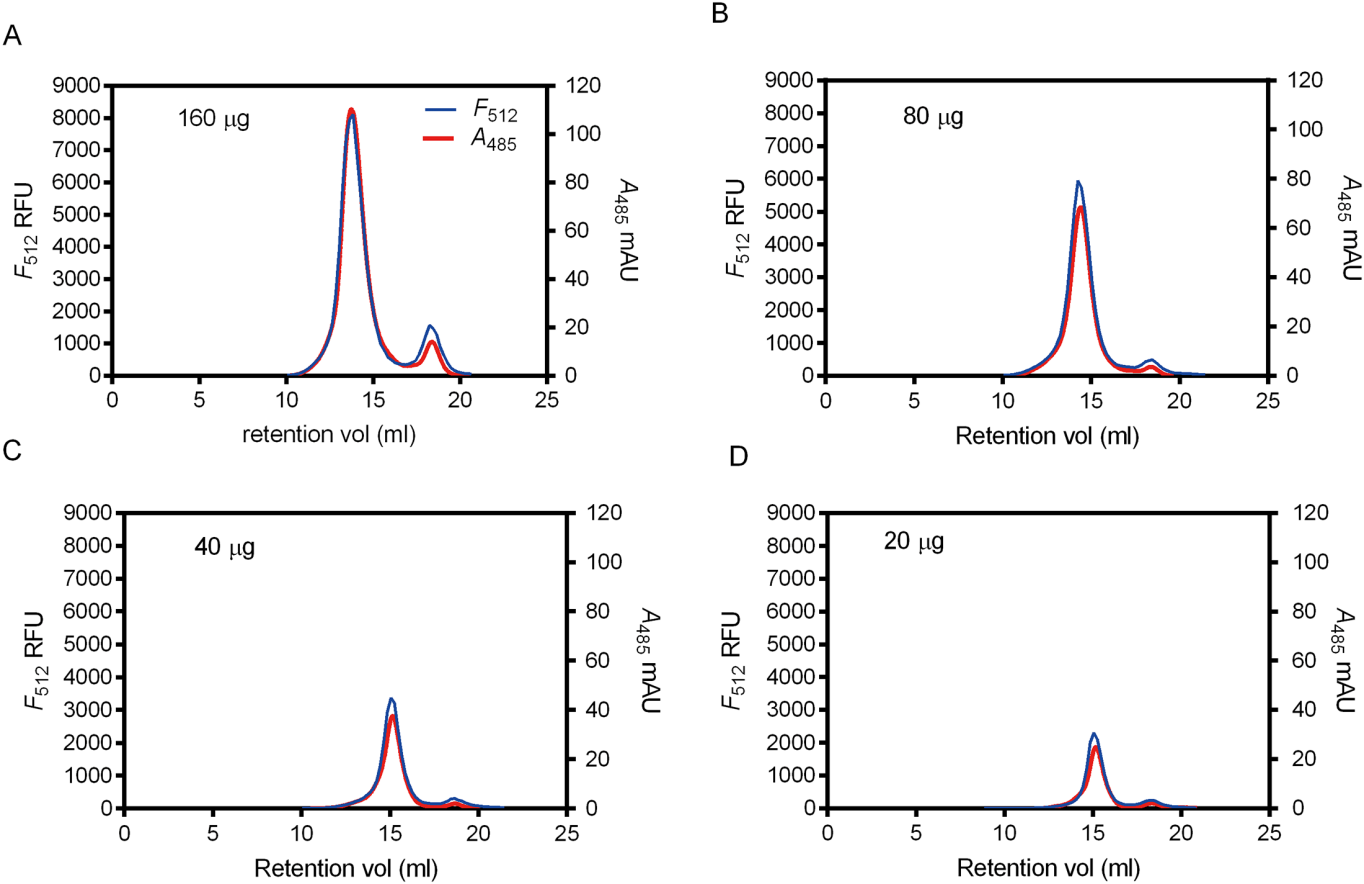
Comparisons of FSEC (blue trace) and FA-SEC (red trace) profiles of ASBT_NM_-EGFP-His8 DDM-solubilized membranes. DDM-solubilized membranes of ASBT_NM_-EGFP (approximately containing 160, 80, 40, and 20 μg of ASBT_NM_-EGFP-His8 in A, B, C and D) were injected in Superose 6 column for profile comparison. All of the FA-SEC profiles were recorded by 10 mm path-length flow cell. The left y axis represents the *F*_512_ intensity and the right y axis represents the *A*_485_ intensity. The scales for fluorescence and absorption profiles in each graph are adjusted to be identical.

Although the A485 signals in FA-SEC profiles are contributed by the C-terminal EGFP and the His8 tag-associated chromophores as discussed above, the two light-absorbing moieties originate from the target proteins. As a result, the measured absorption or fluorescence signals should be proportional to protein concentration. To validate the assumption, we compared the conventional FSEC profiles plotted by the fluorescence counts (F_512_) acquired by microplate spectrofluorometer with the FA-SEC profiles acquired by in-line absorption detector (A_485_). Using the 10 mm path-length flow-cell, we recorded the FA-SEC profiles of different serial diluted DDM-solubilized crude membranes (Fig 3). The comparison of the FSEC and FA-SEC profiles shows a good agreement of peak symmetry and retention volume between the two methods. Moreover, the absorption-emission correlation analysis demonstrates that A485 is proportional to F512 (Fig 2B), validating the feasibility of FA-SEC profiles for detergent screening. Remarkably, as shown in the FA-SEC profiles of serial diluted samples, lower quantity of ASBT_NM_-EGFP (down to 20 μg) does not compromise the data quality and sensitivity (Fig 3D and S1C Fig). It is worth noting that FA-SEC profiles are smooth but FSEC profiles are discontinuous, because, in FSEC experiments, the number of data points is limited using the 96-well microplate reader.

**Fig 4:**
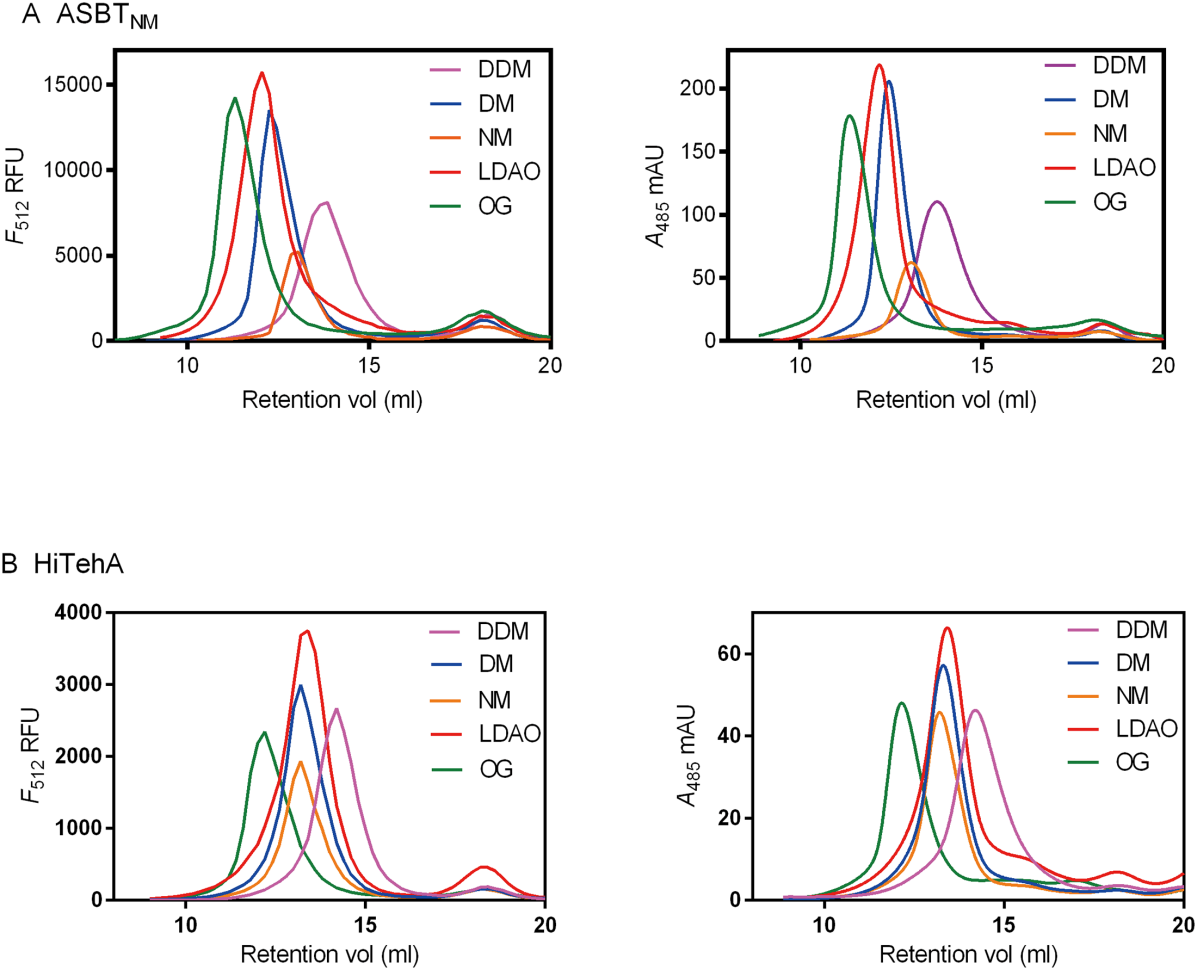
Comparison of FSEC (left panel) and FA-SEC (right panel) profiles of target membrane proteins. (A) ASBT_NM_-EGFP-His8 and (B) HiTehA-EGFP-His8 crude membranes were solubilized in selected detergents (final concentration 1% DDM, 1% DM, 1% NM, 1% LDAO or 2% OG). The FA-SEC profiles were recorded by 10 mm path-length flow cell.

We conducted a detergent screening for monodispersity characterization using crude membranes of ASBT_NM_-EGFP-His8 and HiTehA-His8, and compared the profiles of FA-SEC with FSEC. We tested non-ionic and zwitterionic detergents, including DDM, DM, NM, OG and LDAO, which are commonly used to purify and crystallize membrane proteins (Fig 4 and S4 Fig). For all tested detergents, FA-SEC and FSEC profiles of ASBT_NM_-EGFP-His8 and HiTehA-His8 are highly comparable. Notably, in ASBT_NM_-EGFP-His8 samples, the LDAO-solubilized membranes gave the sharpest and most symmetric peak with the highest height in both traces, corresponding to the fact that the solved structure of ASBT_NM_ was purified and crystallized in LDAO [14]. In HiTehA-EGFP-His8 samples, the peak height is less than ASBT_NM_-EGFP-His8 due to the lower expression level. The crystal structure of HiTehA was determined using OG-solubilized HiTehA [16], also consistent with the results of FA-SEC profiles. Overall, these findings further validated that FA-SEC can be an alternative experimental setup for detergent screening. It is noted that the FA-SEC profiles show smoother traces (Fig 4B) than off-line FSEC (Fig 4A) because the in-line absorption detector produces continuous *A*_485_ readouts.

## Discussion

Based on the strong positive correlation between fluorescence and absorption, and similar profiles compared to the conventional FSEC, FA-SEC provides an alternative approach for detergent screening of membrane proteins. However, specificity and sensitivity of A485 absorption of the new methodology are of potential concern. For non-purified samples like crude membranes, the first concern that one needs to keep in mind is whether contaminants other than EGFP fused target proteins may absorb light at 485 nm and give rise to non-specific background signal. The interfering absorption may originate from intrinsic biomolecules or extrinsic chemicals. We observed the intrinsic A485 signals in the FA-SEC profiles of ASBT_NM_-His8 and HiTehA-His8 membranes. Nevertheless, the background A485 peaks shift in correspondence with the A485 peaks of EGFP-containing counterparts in terms of retention volume in most of the tested detergents (S3 Fig), suggesting the background A485 signals are concomitant with the overexpressed target proteins. It is also shown that the intrinsic background signals were probably coupled to the His-tag because they were eliminated while the His-tag was cleaved (S2A Fig). As a result, both of the detected signals in FA-SEC and FSEC profiles originate from the EGFP-His8-containing target proteins per se, and theoretically they should reach agreement. This assumption can be further validated by the good agreement of FA-SEC and FSEC profiles of ASBT_NM_-EGFP-His8 and HiTehA-EGFP-His8 membranes, demonstrating that the background A485 signals do not interfere in a substantial manner with data analysis (S4 Fig).

In respect of the extrinsic contaminants that may disrupt FA-SEC profiles, they are normally added in the process of sample preparation. For example, Triton X-100, often used to extract membrane proteins for biochemical assays, contains a phenyl group and absorbs UV at λ_max_ = 275 nm [22]. In this study, we tested several detergents commonly used for membrane protein crystallization and the FA-SEC profiles are barely affected compared with the FSEC profiles. We did not test Triton X-100 because it is not useful for membrane protein crystallization due to its heterogeneity.

The second concern of FA-SEC is that the sensitivity of absorption. Fluorescence is 1000 times higher than absorption. This is because fluorescence is measured under low background noise. In contrast, absorption is calculated by difference of two intense signals: the incident light from beam source and the transmitted light. In the study we demonstrated the UV spectrophotometer can detect ~20μg of ASBT_NM_-EGFP-His8 (roughly 10 μg of EGFP). In contrast, the minimum fluorescence signal that fluorometer can obtain is ~10 ng [4]. However, using larger flow cells, we extended the detection limit without affecting FA-SEC profile characteristics, such as peak symmetry, monodispersity, and retention volume (Figs 2, 3 and S1 Fig).

In the structural genomics era, the throughput of structure determination, especially for membrane proteins, require a robust and efficient screening strategy to monitor protein quality at each checkpoint. GFP-fusion is a very versatile strategy for many applications, such as protein over-expression, protein localization, folding, dynamics, stability and protein interactions. FSEC is one of the applications for detergent screening in order to find the most stabilized protein-detergent complex. Conventional FSEC profiles are plotted either using off-line microplate spectrofluorometer or in-line fluorescence detector. The former experimental setup is low-throughput and the profiles are plotted discontinuously, whereas the latter one is not always available in general laboratories. We demonstrated an alternative option to record the SEC profile of non-purified EGFP-fused membrane proteins using FA-SEC. The absorption profiles of FA-SEC are monitored continuously using the in-line multi-wavelength UV detector, which speeds up the throughput compared to the conventional experimental setup using microplates. Additionally, an autosampler can be used in tandem with the HPLC system. This setup facilitates the screening process with even higher throughput and makes it comparable with the UHPLC system (Shimadzu). Moreover, FA-SEC can theoretically be applied to the modified FSEC strategies as mentioned above, such as TS-FSEC, MC-FSEC and fluorescent NTA probe.

## Supporting Information

S1 Fig Signal enhancement of EGFP absorption using 10 mm path-length flow cell. FA-SEC profiles of DDM-solubilized membranes containing approximately (A) 80μg, (B) 40 μg, and (C) 20 μg of ASBT_NM_-EGFP-His8 acquired by 2 (green traces) and 10 (blue traces) mm flow-cells. The normalized profiles (red traces) are plotted using the A485 in FA-SEC profiles of 2 mm flow cell multiplied by 5.
(TIF)

S2 Fig Background A485 signal detected in the FA-SEC profiles of DDM-solubilized membranes. (A) FA-SEC profiles of DDM-solubilized membranes from *E*. *coli* transformed with pET28a(+) (red trace), pASBT_NM_-His8 (blue trace), and pHiTehA-His8 (green trace). The FA-SEC profile of 25 μg purified and tagless ASBT_NM_ is also presented (cyan trace). All of the crude membranes were adjusted to 8 mg ml^-1^ before detergent solubilization and injected membranes contained approximately 15~30 μg of target proteins analyzed by the densitometry of S2B Fig. (B) Immunoblotting of protein samples using anti-His antibody. The purified EGFP-His6 (0.3 μg) soluble protein is a positive control (Lane1). The remaining samples are DDM-solubilized membranes where the crude membranes were adjusted to total protein concentration of 8 mg ml^-1^. 10 μl of DDM-solubilized supernatant was loaded in each well (Lane 2–6).
(TIF)

S3 Fig Comparison of FA-SEC profiles of membranes containing target proteins with or without EGFP. (A) ASBT_NM_-EGFP-His8 and ASBT_NM_-His8, and (B) HiTehA-EGFP-His8 and HiTehA-His8 were solubilized in selected detergents (final concentration 1% DDM, 1% DM, 1% NM, 1% LDAO or 2% OG). The red traces are detergent-solubilized membranes containing EGFP and the blue traces are those without EGFP. The scales for in each graph are adjusted to be identical.
(TIF)

S4 Fig FSEC and FA-SEC profile comparisons of membranes containing target proteins. (A) ASBT_NM_-EGFP-His8 and (B) HiTehA-EGFP-His8 were solubilized in selected detergents (final concentration 1% DDM, 1% DM, 1% NM, 1% LDAO or 2% OG). The left y axis represents the *F*_512_ intensity and the right y axis represents the *A*_485_ intensity. The scales for fluorescence and absorption profiles in each graph are adjusted to be identical.
(TIF)

## References

[1] Deisenhofer J, Michel H. The Photosynthetic Reaction Center from the Purple Bacterium Rhodopseudomonas-Viridis. Science. 1989;245(4925):1463–73. 10.1126/science.245.4925.1463 WOS:A1989AR64800034. 17776797

[2] Drew DE, von Heijne G, Nordlund P, de Gier JW. Green fluorescent protein as an indicator to monitor membrane protein overexpression in Escherichia coli. FEBS Lett. 2001;507(2):220–4. . 11684102 10.1016/s0014-5793(01)02980-5

[3] Drew D, Lerch M, Kunji E, Slotboom DJ, de Gier JW. Optimization of membrane protein overexpression and purification using GFP fusions. Nat Methods. 2006;3(4):303–13. 10.1038/nmeth0406-303 . 16554836

[4] Kawate T, Gouaux E. Fluorescence-detection size-exclusion chromatography for precrystallization screening of integral membrane proteins. Structure. 2006;14(4):673–81. Epub 2006/04/18. 10.1016/j.str.2006.01.013 . 16615909

[5] Newstead S, Kim H, von Heijne G, Iwata S, Drew D. High-throughput fluorescent-based optimization of eukaryotic membrane protein overexpression and purification in Saccharomyces cerevisiae. Proc Natl Acad Sci U S A. 2007;104(35):13936–41. Epub 2007/08/22. 0704546104 [pii] 10.1073/pnas.0704546104 17709746 PMC1955786

[6] Drew D, Newstead S, Sonoda Y, Kim H, von Heijne G, Iwata S. GFP-based optimization scheme for the overexpression and purification of eukaryotic membrane proteins in Saccharomyces cerevisiae. Nature protocols. 2008;3(5):784–98. 10.1038/nprot.2008.44 18451787 PMC2744353

[7] Mizutani K, Yoshioka S, Mizutani Y, Iwata S, Mikami B. High-throughput construction of expression system using yeast Pichia pastoris, and its application to membrane proteins. Protein expression and purification. 2011;77(1):1–8. 10.1016/j.pep.2010.12.009 . 21172439

[8] Brooks CL, Morrison M, Joanne Lemieux M. Rapid expression screening of eukaryotic membrane proteins in Pichia pastoris. Protein science: a publication of the Protein Society. 2013;22(4):425–33. 10.1002/pro.2223 23339074 PMC3610048

[9] Chen H, Shaffer PL, Huang X, Rose PE. Rapid screening of membrane protein expression in transiently transfected insect cells. Protein expression and purification. 2013;88(1):134–42. Epub 2012/12/27. 10.1016/j.pep.2012.12.003 . 23268112

[10] Hu NJ, Rada H, Rahman N, Nettleship J, Bird L, Iwata S, et al GFP-based expression screening of membrane proteins in insect cells using the baculovirus system. Methods Mol Biol. 2014;1261:197–209. 10.1007/978-1-4939-2230-7_11 25502201

[11] Hattori M, Hibbs RE, Gouaux E. A fluorescence-detection size-exclusion chromatography-based thermostability assay for membrane protein precrystallization screening. Structure. 2012;20(8):1293–9. Epub 2012/08/14. 10.1016/j.str.2012.06.009 22884106 PMC3441139

[12] Backmark AE, Olivier N, Snijder A, Gordon E, Dekker N, Ferguson AD. Fluorescent probe for high-throughput screening of membrane protein expression. Protein science: a publication of the Protein Society. 2013;22(8):1124–32. 10.1002/pro.2297 23776061 PMC3832049

[13] Parcej D, Guntrum R, Schmidt S, Hinz A, Tampe R. Multicolour fluorescence-detection size-exclusion chromatography for structural genomics of membrane multiprotein complexes. PloS one. 2013;8(6):e67112 10.1371/journal.pone.0067112 23825631 PMC3692423

[14] Waldo GS, Standish BM, Berendzen J, Terwilliger TC. Rapid protein-folding assay using green fluorescent protein. Nat Biotechnol. 1999;17(7):691–5. 10.1038/10904 . 10404163

[15] Hu NJ, Iwata S, Cameron AD, Drew D. Crystal structure of a bacterial homologue of the bile acid sodium symporter ASBT. Nature. 2011;478(7369):408–11. Epub 2011/10/07. 10.1038/nature10450 21976025 PMC3198845

[16] Chen YH, Hu L, Punta M, Bruni R, Hillerich B, Kloss B, et al Homologue structure of the SLAC1 anion channel for closing stomata in leaves. Nature. 2010;467(7319):1074–80. 10.1038/nature09487 20981093 PMC3548404

[17] Axford D, Foadi J, Hu NJ, Choudhury HG, Iwata S, Beis K, et al Structure determination of an integral membrane protein at room temperature from crystals in situ. Acta Crystallogr D Biol Crystallogr. 2015;71(Pt 6):1228–37. 10.1107/S139900471500423X 26057664 PMC4461203

[18] Shaner NC, Steinbach PA, Tsien RY. A guide to choosing fluorescent proteins. Nat Methods. 2005;2(12):905–9. 10.1038/nmeth819 . 16299475

[19] Mobius K, Arias-Cartin R, Breckau D, Hannig AL, Riedmann K, Biedendieck R, et al Heme biosynthesis is coupled to electron transport chains for energy generation. Proc Natl Acad Sci U S A. 2010;107(23):10436–41. 10.1073/pnas.1000956107 20484676 PMC2890856

[20] Asher WB, Bren KL. A heme fusion tag for protein affinity purification and quantification. Protein Science. 2010;19(10):1830–9. 10.1002/pro.460 WOS:000282716900003. 20665691 PMC2998719

[21] Owens CP, Du J, Dawson JH, Goulding CW. Characterization of Heme Ligation Properties of Rv0203, a Secreted Heme Binding Protein Involved in Mycobacterium tuberculosis Heme Uptake. Biochemistry. 2012;51(7):1518–31. 10.1021/bi2018305 WOS:000300473400019. 22283334 PMC3299815

[22] Tiller GE, Mueller TJ, Dockter ME, Struve WG. Hydrogenation of triton X-100 eliminates its fluorescence and ultraviolet light absorption while preserving its detergent properties. Analytical biochemistry. 1984;141(1):262–6. . 6496933 10.1016/0003-2697(84)90455-x

