# Supplementary figures and images for: Fluorophore Absorption Size Exclusion Chromatography (FA-SEC): An Alternative Method for High-Throughput Detergent Screening of Membrane Proteins
Source: PLoS One. 2016 Jun 22;11(6):e0157923. doi: 10.1371/journal.pone.0157923 (PMC4917255; doi:10.1371/journal.pone.0157923)

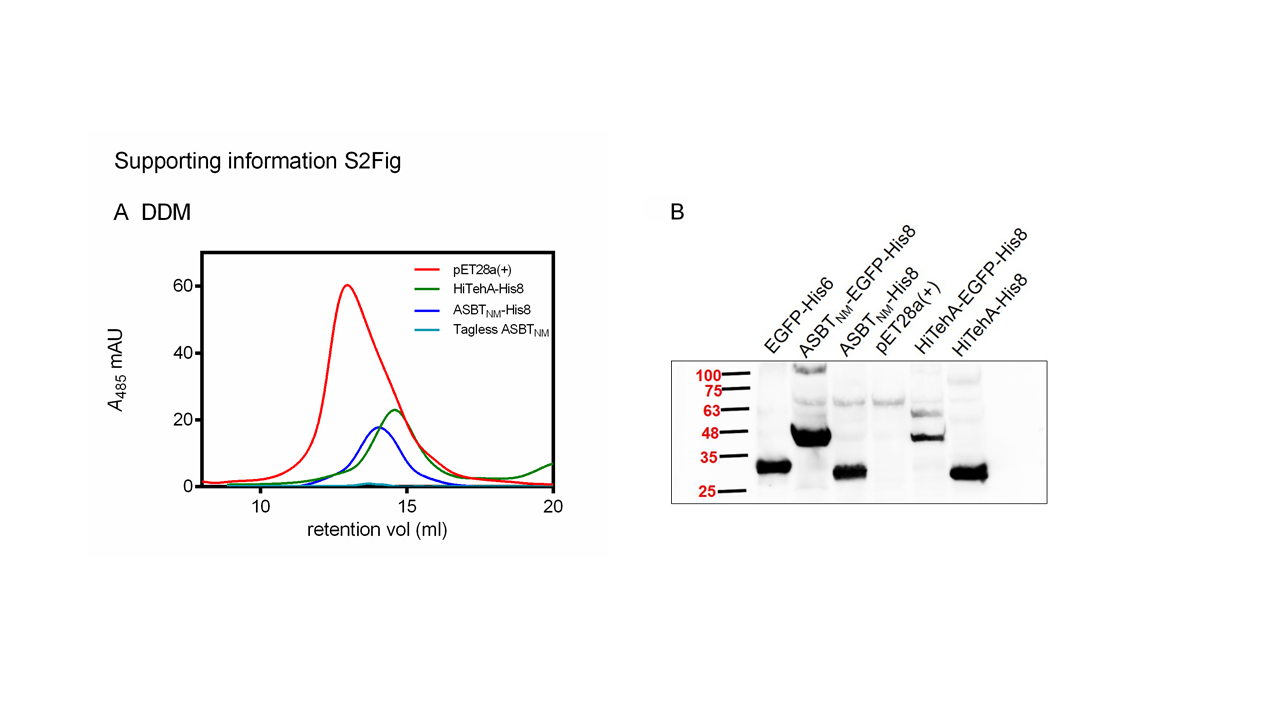

Supplement: S2 Fig — (A) FA-SEC profiles of DDM-solubilized membranes from E. coli transformed with pET28a(+) (red trace), pASBTNM-His8 (blue trace), and pHiTehA-His8 (green trace). The FA-SEC profile of 25 μg purified and tagless ASBTNM is also presented (cyan trace). All of the crude membranes were adjusted to 8 mg ml-1 before detergent solubilization and injected membranes contained approximately 15~30 μg of target proteins analyzed by the densitometry of S2B Fig. (B) Immunoblotting of protein samples using anti-His antibody. The purified EGFP-His6 (0.3 μg) soluble protein is a positive control (Lane1). The remaining samples are DDM-solubilized membranes where the crude membranes were adjusted to total protein concentration of 8 mg ml-1. 10 μl of DDM-solubilized supernatant was loaded in each well (Lane 2–6). (TIF) [file pone.0157923.s002.tif]

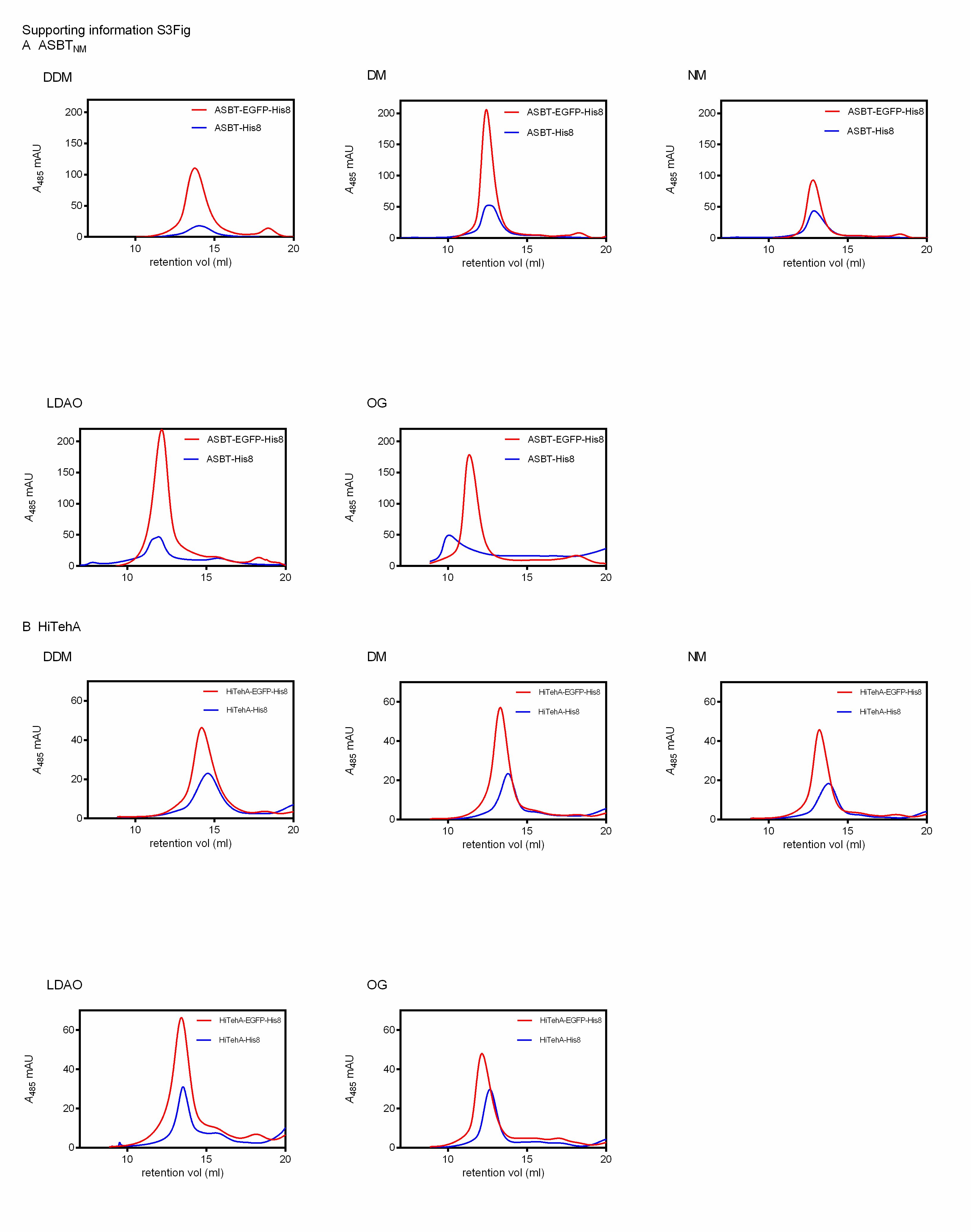

Supplement: S3 Fig — (A) ASBTNM-EGFP-His8 and ASBTNM-His8, and (B) HiTehA-EGFP-His8 and HiTehA-His8 were solubilized in selected detergents (final concentration 1% DDM, 1% DM, 1% NM, 1% LDAO or 2% OG). The red traces are detergent-solubilized membranes containing EGFP and the blue traces are those without EGFP. The scales for in each graph are adjusted to be identical. (TIF) [file pone.0157923.s003.tif]

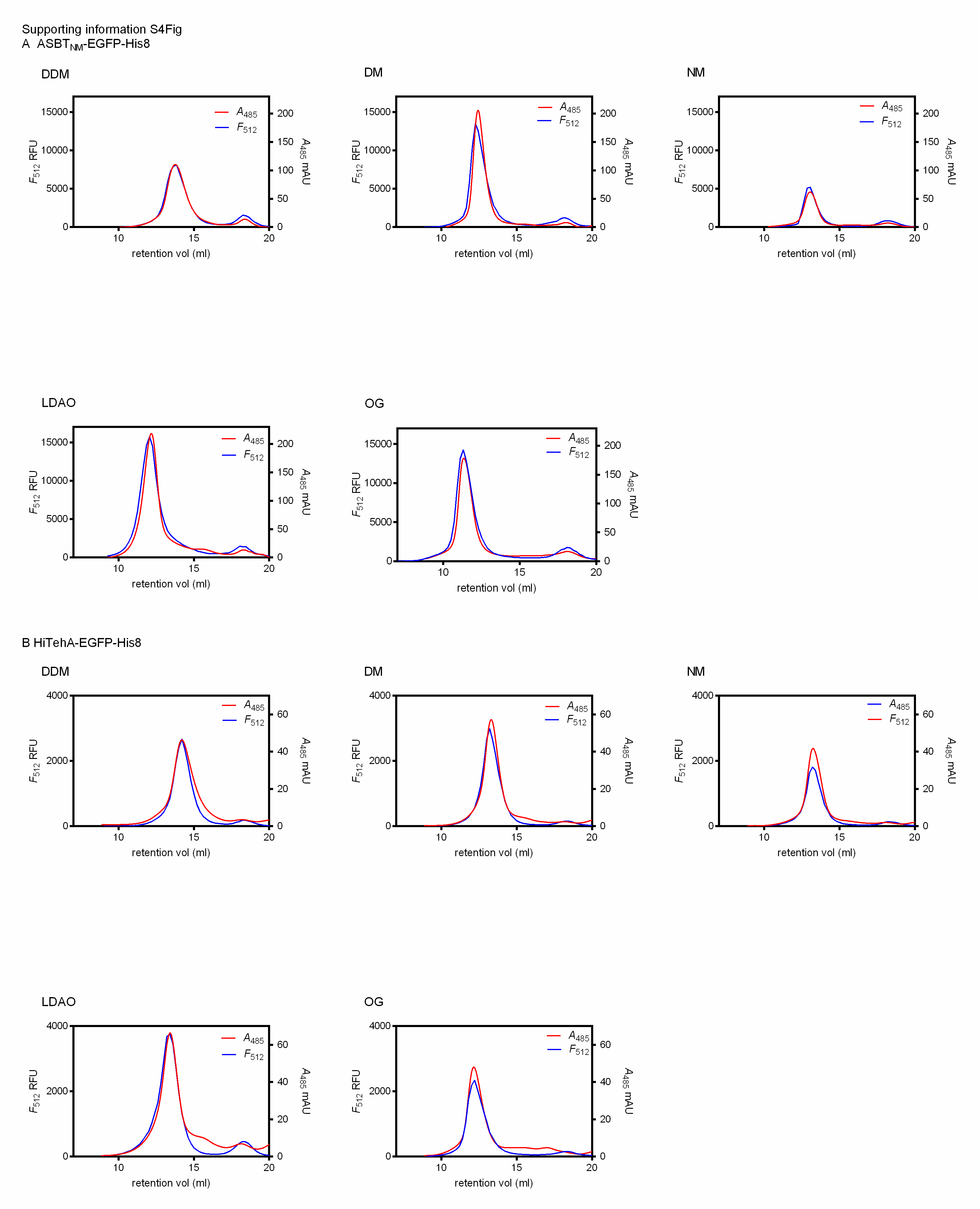

Supplement: S4 Fig — (A) ASBTNM-EGFP-His8 and (B) HiTehA-EGFP-His8 were solubilized in selected detergents (final concentration 1% DDM, 1% DM, 1% NM, 1% LDAO or 2% OG). The left y axis represents the F512 intensity and the right y axis represents the A485 intensity. The scales for fluorescence and absorption profiles in each graph are adjusted to be identical. (TIF) [file pone.0157923.s004.tif]
